# Nano-enabled antimicrobial thin films: design and mechanism of action

**DOI:** 10.1039/d3ra07884a

**Published:** 2024-02-09

**Authors:** Bilisuma Fekadu Finina, Anteneh Kindu Mersha

**Affiliations:** a Department of Industrial Chemistry, Addis Ababa Science and Technology University Addis Ababa Ethiopia; b Nanotechnology Center of Excellence, Addis Ababa Science and Technology University Addis Ababa Ethiopia; c Department of Chemistry, Kotebe University of Education Addis Ababa Ethiopia

## Abstract

Antimicrobial thin films are types of protective coatings that are applied to surfaces such as medical devices, food packaging materials, water-resistant coatings, and other systems. These films prevent and reduce the spread of microbial organisms, including bacteria, fungi, and viruses. Antimicrobial thin films can be prepared from a variety of nanostructured materials including metal nanoparticles, metal oxides, plant materials, enzymes, bacteriocins and polymers. Their antimicrobial mechanism varies mostly based on the types of active agents from which the film is made of. Antimicrobial thin films are becoming increasingly popular microbial treatment methods due to their advantages such as enhanced stability, reduced toxicity levels, extended effectiveness over time and broad spectrum antimicrobial action without side effects on human health or the environment. This popularity and enhanced performance is mainly due to the extended possibility of film designs. Thin films offer convenient formulation methods which makes them suitable for commercial practices aiming at high turnover rates along with residential applications requiring frequent application cycles. This review focuses on recent developments in the possible processing methods and design approaches for assembling the various types of antimicrobial materials into nanostructured thin film-based delivery systems, along with mechanisms of action against microbes.

## Abstract 2

Antimicrobial thin films are types of protective coatings that are applied to surfaces such as medical devices, food packaging materials, water treatment membranes, water-resistant coatings, and other systems.

## Graphical abstract

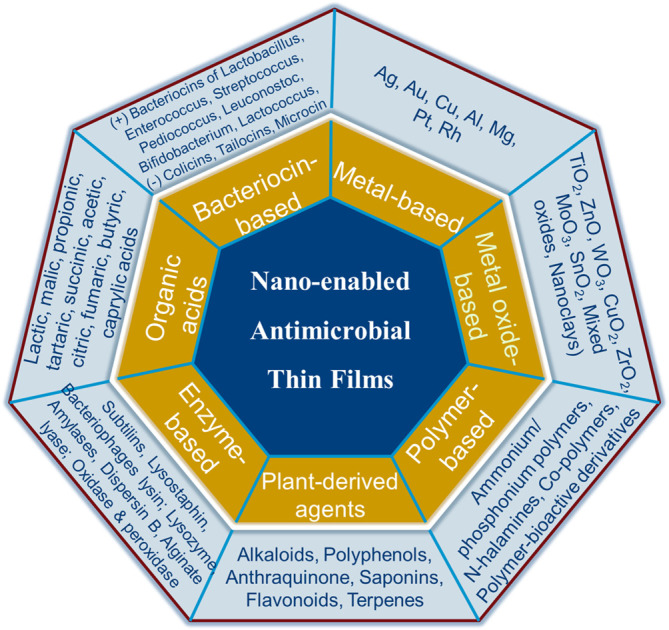

## 1. Introduction

Microbes have become a major concern in health care sectors,^1,2^ water treatment,^3^ food industries,^4,5^ and textile industries.^6^ Establishing an efficient means to control these pathogenic microbes, therefore, has become a primary challenge among the research community.^7^ Use of thin films and other nanotechnology-assisted antimicrobial systems is a viable alternative either to kill bacteria or inhibit the bacterial adhesion to surfaces.^8^ Thin films are believed to be highly convenient drug delivery methods because of their capabilities to improve the onset of drug action, improve target drug delivery, reduce the dose frequency, enhance the drug efficacy, and minimize microbial resistance and side effects to the host.^9^ Membranes, a similar class of flat sheet materials with molecular permeation properties are also excellent platforms to incorporate nanostructured microbicidal agents.^10^

Thin films (TFs), also known as thin-film coatings, are a thin layer of materials applied to a substrate. They typically have a thickness of less than a few hundred nanometers along one direction and extended length along another direction with a large surface area to volume ratio.^11^ TFs and membranes can be supported (*i.e.*, coated on a substrate surface like adhesive coatings) or self-standing like wound dressing breathable films – depending on the preparation method and intended application. Various methods that have their own advantages and disadvantages are employed to prepare TFs and coatings; including simple methods (dip-coating, spin-coating, spray-coating, blade coating, and roll-coating) and those that require sophisticated equipment (such as physical and chemical vapour deposition techniques), which are reviewed elsewhere.^12^

Antimicrobial thin films (ATFs) are thin film structures that possess either inherent or nano-assisted microbicidal property. They are becoming increasingly popular because of their wide array of applications in various fields, mainly in wound dressing, food packaging, textile finishing, and anti-fouling water treatment membranes. ATFs can be used to prevent the growth of harmful microorganisms such as fungi and bacteria on a variety of surfaces through tailoring functionality to target specific microorganisms in various engineered designs.^13^ Designing an ideal antimicrobial thin film requires careful consideration into multitude factors including nature of active agent material, stability against external environmental triggers, manufacturing techniques, geometrical requirements for adherence to substrate material/surface, physical advantages *e.g.*, breathability, suitability for application, release pattern, toxicity limits, exhaustive compatibility against interacting particles and host material.^9^

The mechanism of action behind nano-enabled antimicrobial thin film is that chemical biocides embedded in films are commonly used for anti-microbial action. For instance, cationic residues in thin film commonly first adhere to anionic lipid head groups in the negatively charged membrane surface of bacteria *via* the electrostatic interactions followed by the efficient insertion of their hydrophobic groups into the non-polar bacterial membrane, thereby leading to membrane permeance, cytoplasmic leakage, and bacterial death. Another mechanism of action could be penetration of active agents through microbial cell membranes by oxidation reaction or simply by dissolving into cells *via* electrochemical transduction processes.^14^

In this review, the various classes of nanostructured thin film materials (metallic, metal oxide, polymeric, plant-bioactives, enzymatic, organic acids and bacteriocin) with inherent or nano-enabled antimicrobial properties are discussed. In each class of materials, the research progress on the processing methods and design approaches for assembling antimicrobial agents into functional thin film based delivery systems are presented. Furthermore, the mechanisms of action against microbes, as well as their application in different areas where microbial prevalence is common are addressed.

## 2. General principle of antimicrobial action

**Fig. 1:**
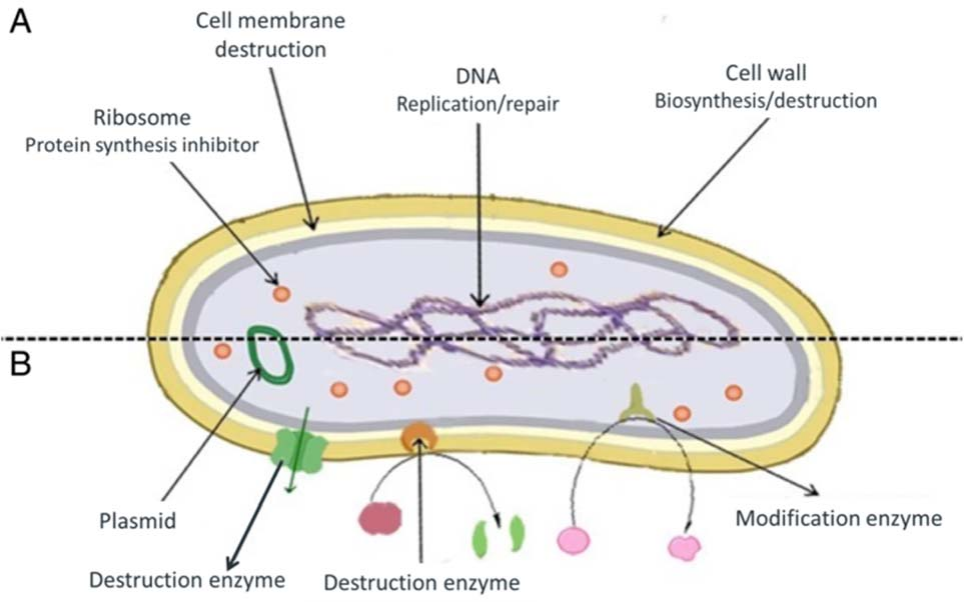
(A) Targets and mechanism for antibacterial action; (B) multiple antibiotic resistance mechanisms in bacteria (reproduced from ref. 16 used under Creative Commons CC-BY license).

Antimicrobials are medicines which are used for treatment or prevention of infections caused by microbes.^15^ The activity of an antibacterial agent is mostly attributed to two mechanisms; interfering chemically with the production or function of essential bacterium components, and/or evading the conventional antibacterial resistance mechanisms. These mechanisms are depicted in Fig. 1, and as can be seen, the antibacterial agents have a variety of targets, including (I) bacterial protein biosynthesis; (II) bacterial cell-wall biosynthesis; (III) bacterial cell membrane destruction; (IV) bacterial DNA replication and repair, and (V) inhibition of a metabolic pathway.^16^Table 1 also provides a summary of the several antimicrobial activity mechanisms along with examples of commercial antibiotics that work according to each mechanism.

**Table 1:** Summary of antimicrobial activity mechanisms and targets

Mechanism of action	Antimicrobial targets	Commercial antibiotics (active *via* same mechanism)	Ref.
Inhibition of protein synthesis	Bacterial ribosome	Macrolides, tetracyclines, aminoglycosides, and oxazolidinones	17
Inhibition of bacterial cell-wall biosynthesis	Transglycosylases and transpeptidases enzymes	Beta-lactams (like penicillins, ampicillins, cephalosporins)	18
	Peptidoglycan layer within cell-wall	Vancomycin	17
Bacterial cell membrane destruction	Bacterial outer membrane permeability	Polymyxins (polymyxin B and colistin)	19
Inhibition of DNA replication and repair	DNA gyrase enzyme	Fluoroquinolones, nalidixic acid	20
Inhibition of metabolic pathway	Folic acid pathway (dihydropteroate synthase enzyme)	Sulfonamides	21

Although antibiotics have historically played a vital role in the prevention and treatment of bacterial infections, there is growing concern that many pathogens develop antimicrobial resistance (AMR), which poses a serious challenge to conventional antibiotic therapies.^14^ AMR can occur in two ways; intrinsic or acquired. Some types of bacteria have an intrinsic resistance to one or more classes of antimicrobial agents. In these situations, all strain of that bacterial species demonstrate resistance to every member of those classes of antimicrobials. The second and more pressing concern is that bacteria might acquire resistance, where previously vulnerable bacterial populations would start to develop resistance to a certain antibacterial agent.^19^ Acquired resistance may result from excessive or frequent use of antibiotics.

The possible mechanism for AMR development includes the following pathways;^14,19,22^ (a) reduced permeability of the bacterial cell wall, restricting antibiotics access to the target sites, (b) overexpression of efflux pumps that actively expels the ingested antibiotics from the bacteria, (c) enzymatic destruction (like amidases, epoxidases, and esterases which are secreted by the bacteria) of the antibiotics to inactivate the antibiotic molecule, (d) alteration of antibiotic targets to avoid the antibiotic binding with the target site or decrease the affinity of antibiotics toward target proteins, (e) altering metabolic pathways of bacteria to dampen the efficacy of antibiotics, and (f) transfer of AMR genes within components in a biofilm *via* quorum sensing.^23–29^ It should be noted that antibacterial resistance might relate to a single mechanism or a combination of the different mechanisms.^19^

## 3. Nano-enabled antimicrobial thin films: classes, design approaches and mechanisms of action

The growing concern of multidrug-resistant bacteria and subsequent diminished effectiveness of current antibiotics brought an urgent need to either develop new antibacterial agents that are less adaptable to microbial resistance or to restrain bacterial resistance using nano-enabled combinatorial therapy with existing antibiotics. Nanotechnology has proven to offer innovative tools in the design and fabrication of high-performance nanostructured delivery systems for antibacterial therapeutic applications.^30,31^

Nano-enabled ATFs are materials incorporated with nanostructured formulations that have the ability to inhibit microbial growth by themselves or trigger the inhibition of bacterial growth by antibiotics. In the former case, nanostructured materials incorporated in thin films generate antimicrobial active agents such as reactive metal ions, photocatalysts and phytochemicals^32,33^ which can directly interact with bacteria. In the latter case, smart nanomaterials assembled in thin films (in the form of encapsulation coatings or precursor agents) improve potency of antibiotic drugs *via* effects like controlled release of active agents,^34^ synergistic efficacy enhancement,^35^ targeted antibacterial effects.^36,37^

**Fig. 2:**
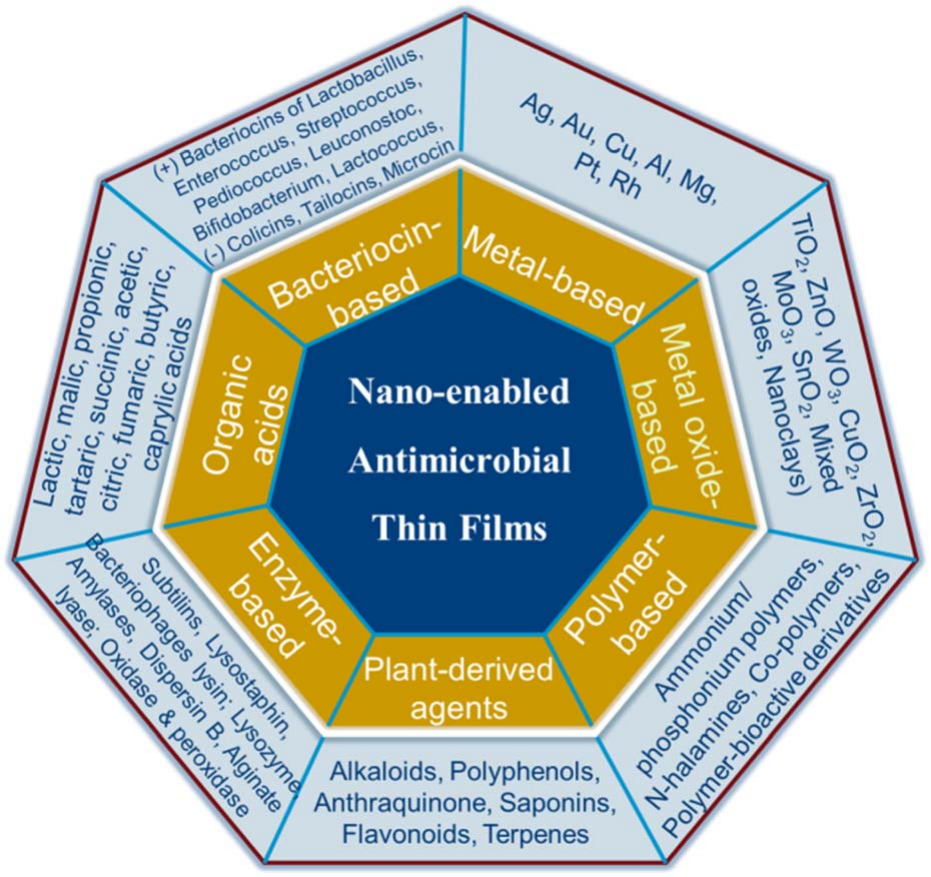
Various classes of nano-enabled antimicrobial thin film systems.

On the basis of their active agents, antimicrobial nanocomposite thin film materials can be classified as: metal-based, metal oxide-based, plant extract-based, enzymes-based, organic acid-based, bacteriocin-based and polymer-based (Fig. 2).

**Fig. 3:**
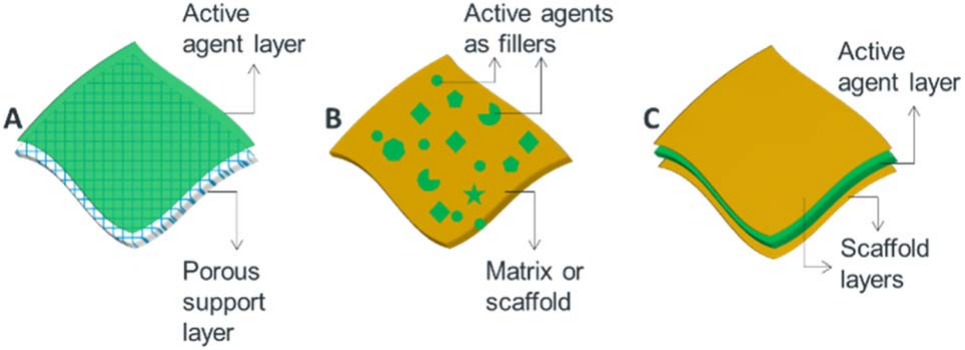
Schematic illustration showing options of incorporating antimicrobial active agents into ATFs: (A) layer or coating approach, (B) blending approach and (C) sandwich structure.

Generally, from the view point of nanostructure design, microbicidal agents can be incorporated into thin films, membranes and surface coatings in three ways. (i) Active agents can be deposited on a porous or nonporous surfaces as a thin upper most layer (Fig. 3A). For example, thin-film nanocomposite membranes^3,38^ which involve the formation of a thin-film layer on a porous polymer matrix, are good examples of such designs. (ii) Blended nano-antimicrobial structures that involve the inclusion of active agents directly into the matrix material are another alternative designs (Fig. 3B). Such structures allow stable incorporation of nano-antimicrobials as the active agents are held in the matrix material leading to reduced leakage.^39^ Mix matrix membranes^40^ and tissue implants^41^ are suitable examples. (iii) The third type of design involves sandwich structures in which active agents are held between two layer structures (Fig. 3C). Such architectures enable controlled and prolonged release of antimicrobial agents, and are suitable to produce wound dressings^42^ and active food packing films.^43^

In the following sections, the possible design alternatives of each class of ATFs from the view point of the way active agents are incorporated and immobilized into composite thin film materials and their antimicrobial mechanisms are summarized in Table 3. It should be noted that the discussions on ATFs also involve antimicrobial membranes-considering that the physical meaning of the term thin film encloses membranes as both materials belong to flat sheet structures.

### 3.1 Metal-based ATFs

Metal nanoparticles are among widely reported materials of promising antimicrobial activity due to their small size and the high surface area-to-volume ratio which gives them a relatively large reactive surface area with which to interact with microbial molecules.^44,45^ They can be easily complexed with other biomaterials to exert enhanced antibacterial activity.^46^ For example, thin films based on silver nanoparticles (AgNPs) and gold nanoparticles (AuNPs) are reported to have high antibacterial activity against different bacterial species.^47^ Copper is another metal known for its good antimicrobial activity with as high as 99.9% bactericidal efficacy.^48^ Cobalt, indium, tungsten, tin, aluminum, chromium, zinc, manganese, tantalum, and titanium-based thin films are also reported to have good antimicrobial activity.^49^ Some of the aforementioned metal-based ATFs are discussed in this section.

Silver nanoparticle based thin film nanocomposite (TFN) membranes prepared by incorporating AgNPs in high surface area graphene oxide (GO) quantum dot and integrated with highly stable polyamide layer.^3^ In this design, suitable amount of AgNPs and graphene oxide quantum dot mixture is sonicated to get homogenized mixture followed by deposition of the mixture on the previously prepared polyamide membrane surface to undergo polymerization. The prepared thin film membrane experienced high antibacterial activity against both Gram-negative (98.6%) and Gram-positive (96.5%) bacteria due to synergetic effect and high surface area of graphene oxide quantum dot (GOQD) occupied by AgNPs. It was demonstrated that antibacterial activity appeared in the order of TFC < TFN-GO < TFN-GOQD < TFN-GOQD/Ag membrane, signifying the importance of Ag loading and nanostructure size, which subsequently increased surface area and amount of reactive oxygen species (ROS) generation.

The authors also studied the bactericidal mechanism for GOQD/Ag, where the restriction to respiratory enzymes by AgNPs induced the release of ROS through the oxygenolysis of the cellular components. ROS could oxidize the lipids in the bacterial cell membrane, disturbing the cell metabolism and leading to cell death. They further justified that the high oxidase-like catalytic activity of GOQD/Ag induced an outstanding antibacterial property of the TFN-GOQD/Ag membrane *via* increased ROS generation.

**Fig. 4:**
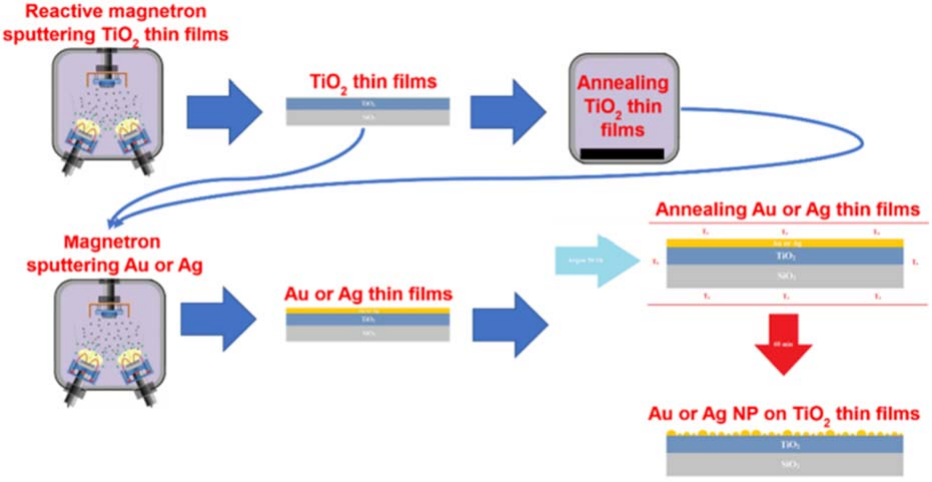
Formation of AuNPs/AgNPs-TiO_2_ thin film (reproduced from ref. 47 used under Creative Commons CC-BY license).

In another study, Qingquan. G *et al.*^50^ designed polymeric thin film membrane decorated with ultra-small silver nano-cluster (AgNCs) encapsulated in layer of thiolate ligands. Fig. 4 shows the fabricated thin film with highly stable AgNCs-thiolate ligands complex deposited on polymeric membrane surface. The strong bonding between thiolate ligands and AgNCs could be responsible for slow-release of Ag ions, which would further regulate the concentration of Ag ions on the film surface at a constant level. This process resulted in the long life time, tunable and sustainable antibacterial behaviour of the AgNC-modified thin-film composite membrane.

**Fig. 5:**
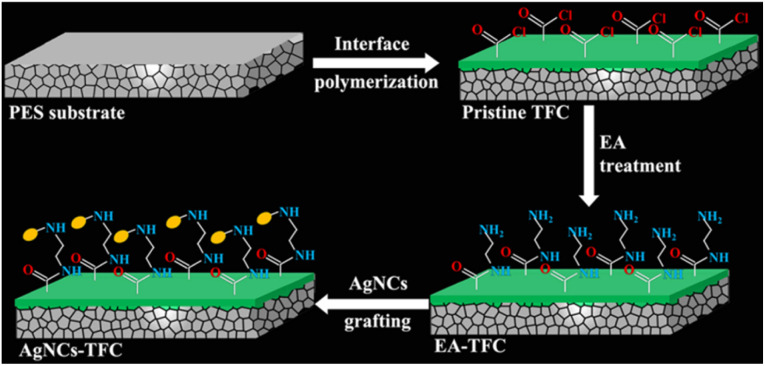
Schematic illustration of the fabrication of AgNC-modified thin-film composite (AgNC-TFC) membrane (reproduced from ref. 50 with permission from American Chemical Society).

Gold is another noble metal used to prepare bactericidal thin film on the surface of different materials including medical devices due to its low toxicity and a great bio-affinity. Villa Garcia L. *et al.*^51^ reported coating (*via* magnetron sputtering) of gold nanoparticles on polyethylene, a versatile polymeric material used in surgical instruments to temporary and permanent biomedical devices. The gold coated polyethylene demonstrated strong biofilm inhibition activity. In another work, titania thin films decorated with AuNPs and AgNPs also demonstrated higher antibacterial activity. Briefly, amorphous TiO_2_ thin film is prepared by using magnetron sputtering method and crystallized by annealing at high temperature. Consequently, AuNPs and AgNPs are deposited onto the crystallized TiO_2_ thin film under vacuum condition followed by annealing at high temperature.^47^Fig. 5 shows the designed titania thin film coated with AuNPs and AgNPs.

T. Kruk, *et al.*^32^ reported copper nanoparticles (CuNPs) suspension coated poly(diallyldimethylammonium chloride) multilayer antibacterial film. Their result indicated the developed multilayer composite film coated with CuNPs showed significant decrease in the cell viability of *S. aureus* bacteria after 6 hours of incubation.

The primary mechanism by which metal-based thin films can inhibit microbial growth is through the release of ions such as Ag^+^, Zn^2+^, Au^+^ and Cu^2+^.^33^ These ions directly interact with cell walls of microorganisms to create pores or holes that disrupt normal functioning. In addition, they also form complexes with enzymes or other cellular components that can further inhibit biofilm growth or cause membrane damage and cell death. For example, silver ions have been found to be able to penetrate bacterial cell membranes rapidly, leading to disruption of oxidative processes such as respiration and denaturation of proteins and then damage the integrity of microbial cells. In another work, T. Kruk, *et al.*^32^ confirmed the antibacterial mechanism of CuNPs is through depolymerisation of bacterial cell membrane when in contact with CuNPs and generation of ROS which in turn cause cellular lipid peroxidation, protein oxidation, DNA degradation; and finally bacterial cell death.

**Fig. 6:**
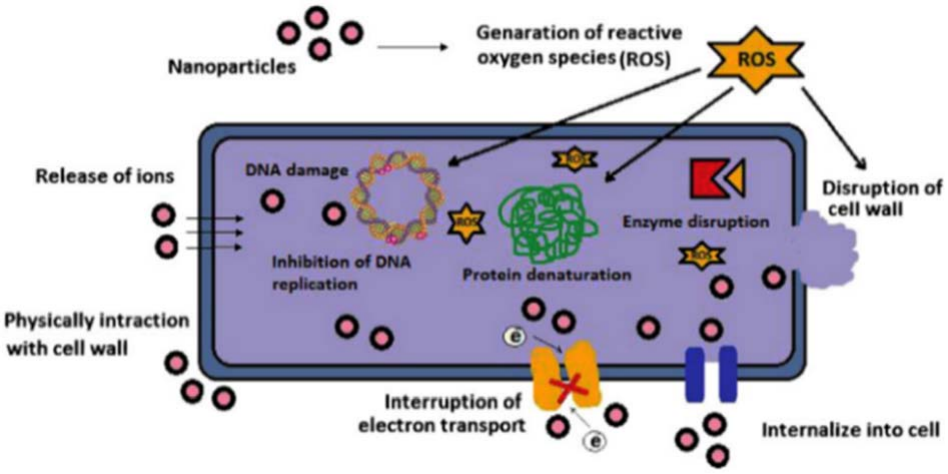
Various mechanisms of antimicrobial activity of metal nanoparticles (reproduced from ref. 45 with permission from Elsevier).

Fig. 6 demonstrates antimicrobial mechanisms of metal nanoparticles based thin film. Accordingly, (1) release of metal ions from the metal nanoparticles and (2) direct interaction of the metal ions and/or (3) metal nanoparticles with the cell wall through electrostatic interactions, leading to impaired membrane function and impaired nutrient assimilation; (4) formation of extracellular and intracellular reactive oxygen species (ROS), and damage of lipids, proteins and DNA by oxidative stress; (5) high-levels of metal-binding to the cell envelope and high ROS levels can cause damage to the plasma membrane and thus lead to the leakage of the cell content; (6, 7) upon metal uptake, metal nanoparticles and metal ions can directly interfere with both proteins and DNA, impairing their function and disturbing the cellular metabolism in addition to metal-mediated ROS production are reported antimicrobial mechanisms of metal nanoparticles.^52^

### 3.2 Metal oxide-based ATFs

Metal oxide (MO_*x*_) semiconductors are among the widely employed antimicrobial materials due to their photocatalytic properties.^53^ Various metal oxide nanomaterials including CuO, ZnO, titania (TiO_2_), alumina (Al_2_O_3_), iron oxides, ZrO_2_, silica (SiO_2_), and others used in the development of antibacterial thin films and membranes.^54^ They are processed into nanocomposite films in various designs and architectures; distributed in a polymer matrix, layered film structures and hybrids.^55^ The frequently reported approach to fabricate antimicrobial MO_*x*_ nanocomposite films is using a MO_*x*_/polymer layered structure,^56^ including layer-by-layer (LBL) alternate deposition of MO_*x*_ and polymer structures.^57^ Due to the versatility of MO_*x*_ nanomaterials, MO_*x*_-based antimicrobial thin films and membranes are extensively applicable in areas such as medical disinfection coatings, water treatment membranes and self-cleaning surfaces.

MO_*x*_ coating materials which have antibacterial property and prevent bacterial adhesion are desirable in implant technology to prevent bacterial infection associated trauma and high cost.^58^ For example, antibacterial agents incorporated mesoporous titania thin films characterized by controlled drug release, high pore volume and high surface area which make them ideal drug loading site is among the promising materials which can be coated onto the medical devices.^59^ Atefyekta *et al.* reported titanium oxide-based mesoporous titania thin film loaded with the antimicrobial agents (vancomycin, gentamicin, and daptomycin) in its pore volume. The thin film is characterized by decreased biofilm attachment to its surface and tunable antibacterial loading observed to be increased with increasing pore volume; thus highly contribute to host tissue growth and reduced biomaterial associated infections.^60^

**Fig. 7:**
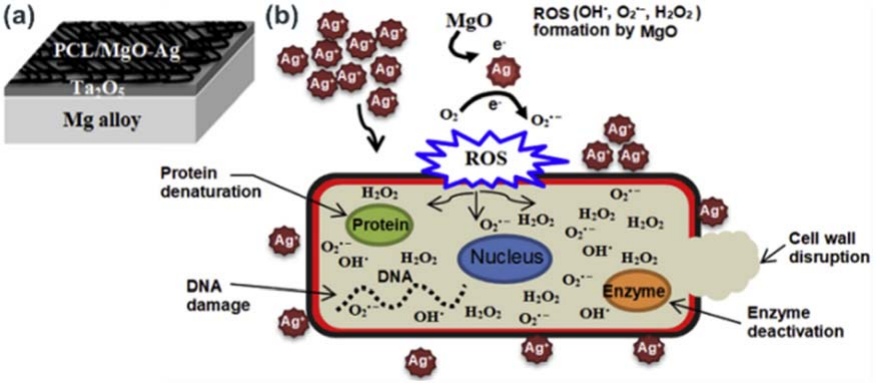
A schematic representations of (a) electrospun nanofiber coatings and (b) antimicrobial mechanisms of PCL/MgO–Ag (reproduced from ref. 61 with permission from Elsevier).

In another study, orthopedic implant has been developed by subsequent deposition of tantalium dioxide (Ta_2_O_5_) and poly(ε-caprolactone)/magnesium oxide–silver (PCL/MgO–Ag) nanofiber porous layers on Mg alloys.^61^ In this work Ta_2_O_5_ was coated on Mg alloys *via* magnetron sputtering using argon gas as the sputtering gas. Then, homogenised PCL/MgO–Ag film forming solution is deposited onto the previously prepared Mg alloy-Ta_2_O_5_ layers (Fig. 7a). Antibacterial efficacy test of the developed thin film indicated high antibacterial activity which is supposed to be due to synergetic antibacterial activity of MgO and Ag in the thin film. The antibacterial mechanism of PCL/MgO–Ag is illustrated in Fig. 7b. MgO reacts with intracellular oxygen and generate ROS, such as OH˙, O_2_˙^−^, and H_2_O_2_, which may dissolve AgNPs and subsequently generate Ag^+^ ions into the medium. This synergetic process causes further inhibition of proliferation and bacterial growth *via* cell wall disruption, protein denaturation, enzyme deactivation and DNA damage. The MgO-induced oxidative dissolution of AgNPs into Ag^+^ and successive increase in bactericidal effect was further confirmed by other reports.^62,63^

Thomas D. *et al.*^64^ reported microwave assisted successive layer of iodine doped ZnO thin film. The film was developed by depositing ZnO on indium titanium oxide glass substrate followed by UV irradiation and the process repeated many times. The prepared ZnO is doped by adding iodine solution and annealed at high temperature. The antibacterial test of the thin film indicated that antibacterial activity of thin film is through splitting of the bacterial membrane. This disruption of bacterial membrane results from high surface oxygen species generation from ZnO which leads to the bacterial death. Another report also confirmed that visible light contacted ZnO NPs results in increased H_2_O_2_ which disturb cellular homeostasis and DNA lysis that eventually prompt premature bacterial cell death.^65^

Silver nanoparticle doped TiO_2_ and SiO_2_ double layer thin films deposited on self-adhesive polyurethane foil has been developed. AgNPs doped and double layer thin film showed improved antibacterial activity when compared with non-doped and single layer thin films respectively. In this work, UV light exposure is reported to affect antibacterial efficacy of the thin film, which UV light stimulated thin film experienced enhanced antibacterial activity when compared with non-UV light treated film.^1^

Another prominent area of application for MO_*x*_-based nanostructures is in the development of antimicrobial membranes and surfaces for photocatalytic water disinfection systems.^66,67^ We also have recently reviewed cellulose supported photocatalytic membranes where photoactive MO_*x*_ nanomaterials are discussed. In view of nanostructure design, MO_*x*_ are incorporated as fillers to polymeric membranes to minimize biofilm formation, to kill pathogenic microorganisms, to increase water flux *via* imparting membrane hydrophilicity and pore formation, and to improve mechanical properties.

Chaudhary *et al.*^40^ developed cellulose acetate (CA) based mixed matrix membrane (MMM) using mixed metal oxides nanoparticles-polymer composite (Fe–Al–Mn@chitosan) as nanofiller. The excellent antibacterial property of the MMM was due to the stable incorporation of nano-Fe–Al–Mn@chitosan inside the CA matrix.

**Fig. 8:**
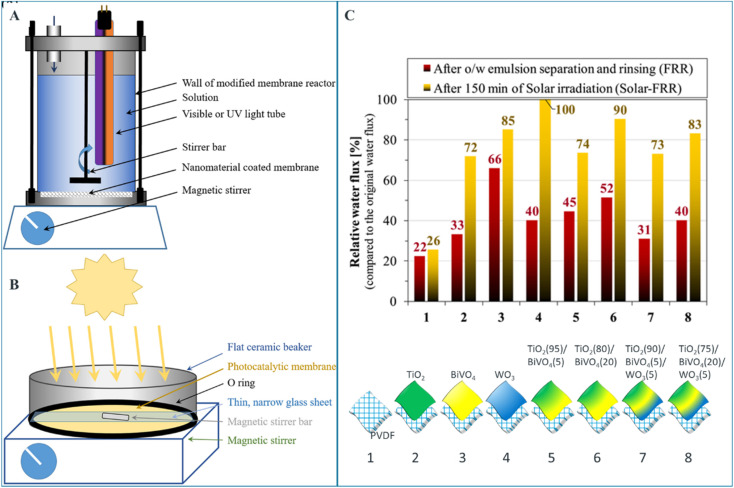
Schematic figure of (A) the photoreactor used for the comparison of photocatalytic activities of different TiO_2_/WO_3_ nanocomposite-covered membranes and (B) the experimental setup used for the solar photocatalytic membrane cleaning experiments, (C) solar photocatalytic flux recoveries after 150 min of solar irradiation of the contaminated membranes (coating compositions are defined under the graph. The numbers in the brackets represent percent composition of the nanocomposites).

Santos *et al.* investigated the photocatalytic activities and subsequent oily water purification performance of PVDF ultrafiltration membrane modified by different metal oxides TiO_2_, BiVO_4_, and WO_3_.^68^ As it can be seen in Fig. 8, upon solar irradiation, the membranes disinfect themselves from biofilms in a chemical-free manner, and sufficiently recover the membrane flux. The authors claim TiO_2_ (80%)/BiVO_4_ (20%) composite coating demonstrated a good compromise between flux and self-cleaning properties.

MO_*x*_ nanocoatings are also employed in thin film supercapacitors to impart antimicrobial surface property coupled with water resistance and transparency. Choi *et al.*^69^ developed zinc aluminate films for smartphone panel glass that is resistant to water and acidic water while demonstrating self-disinfecting and hydrophobic behaviours. The MO_*x*_ thin film with good stability and strong antibacterial activity was prepared by depositing ZnAl_2_O_4_ layer onto gorilla glass as a substrate using radio frequency magnetron sputtering technique. The strong antibacterial activity of the thin film is due to electrostatic interaction between negatively charged bacterial cell wall and high positive surface potential of the ZnAl_2_O_4_ layer onto gorilla glass where the reaction could result in an effective bacterial death.

**Fig. 9:**
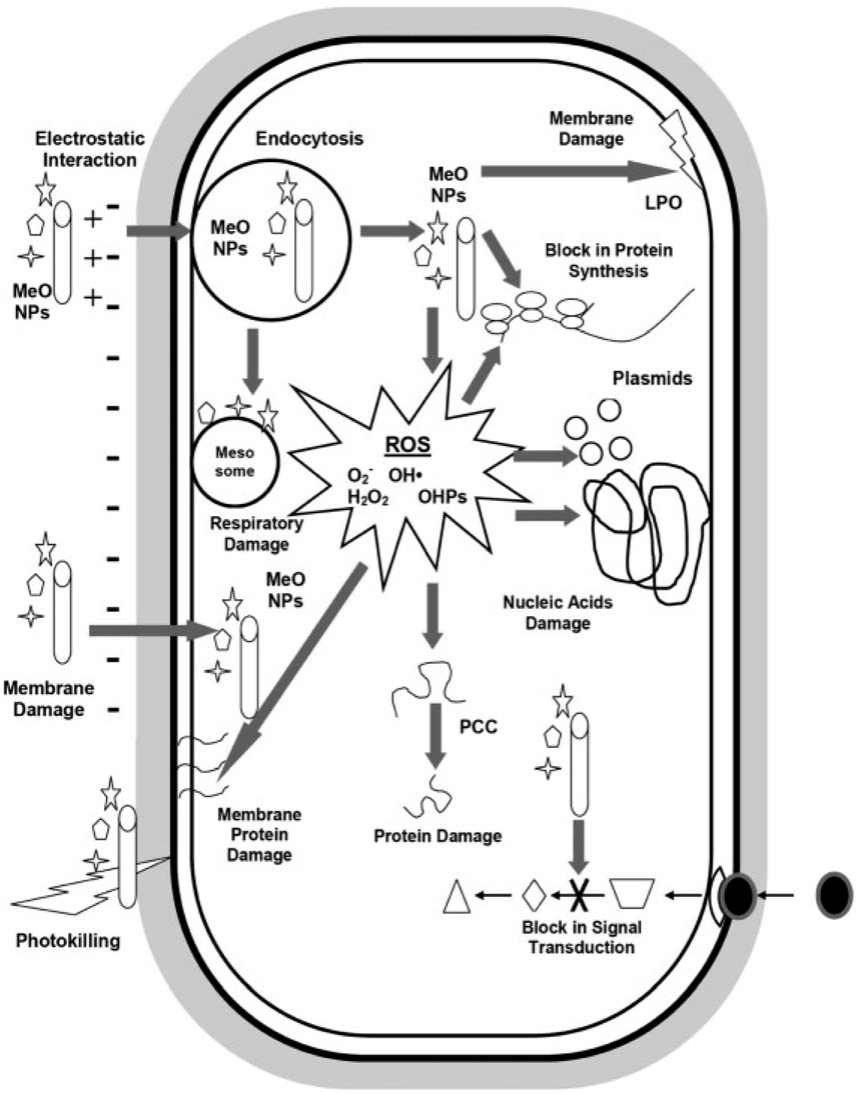
Overview of antimicrobial mechanisms by metal oxide nanoparticles (reproduced from ref. 53 with permission from Elsevier).

In general, the principal antimicrobial mechanisms of metal oxides is generation of ROS like H_2_O_2_, O_2_˙^−^ and OH˙ which increase oxidative stress and cause microbial cell damage.^70^ Another mechanism behind microbial cell damage is due to the electrostatic interaction between metal cation generated from metal oxides and negatively charged microbial membrane which cause destruction of the membrane that allow metal oxides nanoparticles entry to the cell. Once metal oxides nanoparticles entered the microbial cells, they react with biomolecules and cause cell disruption which ends up with microbial death.^53^Fig. 9 shows general antimicrobial mechanisms of metal oxide nanoparticles. Metal oxides also facilitate the controlled release of metal ions from metal nanoparticles, which leads to synergetic bactericidal effect.^71^ It is worth noting that the mechanism of microbial action by MO_*x*_ nanoparticle thin films and membranes vary considerably, requiring further investigations which would help in systematically designing antimicrobial nanostructures of a particular MO_*x*_ on surfaces and functional systems.

### 3.3 Metal organic framework and nanoclay based ATFs

Metal organic frameworks (MOFs) and nanoclays (NCs) are versatile ceramic materials with well-defined porous structures. Thin films and membranes of MOF and NC materials possess inherent antimicrobial properties. Nejad *et al.* reported the preparation of anti-fouling and anti-biofouling zeolitic imidazolate framework-7 on the surface of functionalized porous polyethersulfone substrate.^72^ The inhert antibacterial properties of MOF and clay nanomaterials vary with their nanoparticle size and shape, type of metal atoms and ligands present, and their crystalline structures. Nevertheless, their antibacterial activity is generally a result of their structural collapse,^73^ and subsequent release of metal ions and ligands.

Metal-doped and other functionalized MOF membranes are also widely researched microbicidal materials, with Ag-based MOFs becoming very prominent in the area.^38,74,75^ In such nanocomposite designs, the incorporated metal ions introduce additional activity against microbes. Seyedpour *et al.*^38^ fabricated a high antibacterial and antifouling Ag-MOF nanorod modified thin film nanocomposite (TFN) forward osmosis membranes. They developed the TFN-FO membranes in two steps: synthesis of Ag-MOF nanorods followed by interfacial polymerization of polyamide in the presence of 2-aminoterephthalic acid treated Ag-MOF. Even though the nanorods were buried within the polyamide matrix, they could still produce high bacterial inactivation in a short bacteria-membrane contact time of 1 h.

MOFs and NCs also serve as charge carriers in the development of nanocomposite photocatalysts with semiconductors, resulting in enhanced photocatalytic performance.^76,77^ For example, palygorskite (Pal), the environmentally friendly nanoclay, based membrane has been prepared for reverse osmosis (RO) water desalination. The thin film nanocomposite (TFN-Pal/TiO_2_) RO membrane was designed by incorporating Pal-TiO_2_ nanocomposite in the polyamide (PA) selective layer *via* interfacial polymerization. The membranes exhibited strong photocatalytic antibacterial activity while maintaining high water flux and anti-fouling property in the order of TFN-Pal/TiO_2_ > TFN-Pal > TFC for all antibacterial, water flux and fouling resistance tests. UV treatment of the thin film membrane generated ROS like OH˙ and H_2_O_2_ and these ROS could damage bacterial membrane and block bacterial growth.^76^

### 3.4 Plant-derived ATFs

Long time, synthetic antibiotics were used to treat and prevent microbial invasion to overcome microbial associated risks in different sectors. However, synthetic antibiotics are limited to rapid rise of antimicrobial resistance, their undesired side effect and even death.^78,79^ In this regard, bioactive phytochemicals are being discovered as the viable alternative to overcome or minimize these associated risks.^79,80^ For instance, antimicrobial thin films prepared from plant extracts, essential oils, and active compounds are widely used to control biofilm formation on the contact surfaces. Such activity is contributed from plant-derived secondary metabolites such as alkaloids, polyphenols, flavonoids, tannins, anthraquinone, saponins and terpenoids embedded in a matrix of sterile polymers.^80,81^ Even though there is no clear antimicrobial mechanism of bioactive phytochemicals reported yet, different phytochemicals are suggested to act through different mechanism^82^ as shown in Table 2.

**Table 2:** Suggested predominant antimicrobial mechanism of some plant secondary metabolites

Bioactive compounds	Suggested predominant antimicrobial mechanism of action	Reported plant origin	Ref.
Alkaloids	Efflux pump (bacterial transmembrane protein complexes) inhibition followed by cell membrane destruction	*Callistemon citrinus*, *Vernonia adoensis* and *Peganum harmala*	82 and 83
		*Papaver rhoeas*	84
	Inhibition of bacterial cell wall and intercalating into bacterial DNA	*Papaver glaucum* and *Papaver decaisnei*	83 and 85
Polyphenols	Inhibition of bacterial protein biosynthesis	*Rhus coriaria*, *Viscum cruciatum Sieb* and *Quercus infectoria olive*	86
	Interaction with the bacterial cell wall and cell membrane	Cloudberry, black currant, cranberry, and blueberry extracts	87
		Grape seed, apple, cinnamon bark, and rosemary leaves	88
	Inhibition of metabolic pathway	*Mentha longifolia*, *Gentian lutea*, *Nigella sativa*, *Chamomilla recutita*, *Murraya koenigii*, and *Terminalia chebula*	89
Anthraquinone	Cell wall and membrane disruption	*Curtisia dentate*	90
		*Vismia laurentii*	91
	Binding to bacterial DNA	*Cassia nodosa*	92
Saponins	Cell membrane disruption	*Chenopodium quinoa*	93
		*Camellia oleifera*	94
	Cell wall and cell membrane damaging	Green tea seed	95
Flavonoids	Cell membrane disruption	*Anemarrhenae rhizome*, *Gardeniae fructus*, and *Mangosteen peel*	80 and 96

**Table 3:** Summary of various classes of nano-enabled antimicrobial thin films; their development approaches, and antimicrobial action mechanism^a^

Category	Nano-structure design	Type of material	Film preparation method	Predominant antimicrobial mechanism	Ref.
Metal-based thin film	Layer-by-layer	Thiolete/AgNCs/thiolete	Soaking	Electrostatic interaction and membrane penetration	50
	Single layer	Au/polyethylene	Surface coating		51
	Multilayer deposition	Ag/TiO_2_/polyurethane	Doping		1
Metal oxide-based thin film	Layer-by-layer	ZnO/ZnO/I	UV irradiation	ROS generation & electrostatic attraction	65
	Multilayer deposition	Ta_2_O_5_/(PCL/MgO–Ag)/Mg alloy	Magnetron sputtering (1st layer) and electro-spinning (2nd layer)		61
	Multilayer deposition	(AZO/Ag/AZO)/ZnAl_2_O_4_/PET/(AZO/Ag/AZO)			69
	Surface deposition	WO_3_/stainless steel	Magnetron sputtering		70
Plant-based thin film	Dispersion	CH/ZO	Solution casting	Cell wall/membrane interaction	98
	Dispersion	PC/LE	Solution casting		81
	Dispersion	AVO/CA & RO/CA	Solution casting		100
	Dispersion	GEO/Starch	Solution casting		99
Enzyme-based thin film	Layer-by-layer	Endolysin/SiO_2_	Solution casting	Attack DNA	113
Organic acids-based thin film	Dispersion	API/BA	Solution casting	Lowering intracellular pH	119
	Dispersion	LA or MA/Zein	Solution casting		118
	Dispersion	LA/PVA	Solution casting		120
	Dispersion	CitA/CMC	Solution casting		117
Bacteriocin-based thin film	Dispersion	M35/CH	Solution casting	Cell membrane disruption	130
	Dispersion	Bacteriocin/CNC	Solution casting		132
	Dispersion	Bacteriocin/agar–agar polysaccharide	NS		129
Polymer-based thin film	Layer-by-layer	Zn@CuO/polydopamine	Coating	Prevent adhesion/cell wall interaction/penetration	144

a Abbreviations: PC – polycarbonate, LE – leaf extract, PCL – polycaprolactone, ZO – *Zingiber officinale*, CNC – cellulose nanocrystal, AZO-Al-doped ZnO, PET – polyethylene terephthalate, NS – not specified, AVO – aloe vera essential oil, RO – Rosemary essential oil, CA – cellulose acetate, CitA – citric acid, CMC – carboxymethyl cellulose, BA – benzoic acid, LA – lactic acid, MA – malic acid, API – anchovy protein isolate.

*Zingiber officinale* is among bioactive constituent rich medicinal plant with high antibacterial effect.^97^*Z. officinale* essential oil incorporated chitosan film with enhanced antibacterial activity has been reported.^98^*Z. officinale* essential oil is dispersed in chitosan containing solution to facilitate essential oil–chitosan interaction. The film forming solution is casted on Petri dish and allowed to dry to form the thin film. The interaction holding the film components together is reported to be interaction between the chitosan hydroxyl and amine group with the phenolic compounds *Z. officinale* essential oil. Garlic is another antibacterial rich plant processed in to active packaging film. Sihombing N. *et al.*^99^ prepared garlic essential oil-based edible active packaging by coating essential oil film onto the previously plasticized and moulded cassava starch. The film demonstrated high antibacterial activity against *Escherichia coli* and extended shelf-life. Rosemary and aloe vera oil based cellulose acetate film is another plant extract-based antibacterial thin film. El Fawal *et al.*^100^ developed bioactive packaging membrane from rosemary and aloe vera essential oils emulsified separately into cellulose acetate polymer matrix. The portions of incorporated essential oils are reported to move to the membrane surface during the drying process. Thus the thin film membrane showed strong bactericidal activity against tested bacteria; whereas bare cellulose acetate used as a control does not show any antibacterial activity.

Aaliya B. *et al.*^101^ developed plant extract incorporated active food packaging thin film materials. Plant extracts from neem tree, tulsi, Mexican mint, and curry leaves is mixed with gelatinized carboxymethyl cellulose and glycerol and further homogenized prior to casting. The developed thin film demonstrated excellent antibacterial activity against *Staphylococcus aureus* and *Escherichia coli*.^101^ Polyethylene surface (PE) decorated with different plant extract using surface coating techniques also has been reported. In this work, suitable amount of rosemary, raspberry, and pomegranate CO_2_ extracts is mixed with fixing agent followed by homogenization. PE plate is inserted in the film forming solution, stirred and extruded by twin-screw extruder. The resulted thin film is again extruded through a flat die to obtain films with a uniform thickness. The developed thin film is reported to inhibit the growth of some bacterial strains.^102^ Ali *et al.*^103^ fabricated antimicrobial food packaging thin films incorporated with different medicinal plants (*Acontium heterophyllum*, *Artemisia annua*, and *Thymus serpyllum*) and tested their bactericidal activity against *S. aureus*, and *Salmonella*. The result demonstrated good zone of inhibition against both bacterial strain. Increment in zone of inhibition with increased plant extract concentration was reported in this work.

The interaction between the active molecules of plant-based thin film and microbial cell is the key mechanism by which plant extract exhibit increased antimicrobial efficacy against various classes of microbes. Synergistic activities of plant bioactive constituents also play a major role in providing great protection not only against single species but even cocktail species thus making it more effective compared with individual independent compound approaches.^104^ To the point, these molecules exhibit inhibition mechanisms such as reducing membrane permeability, increasing osmotic pressure, inhibition of nucleic acid and protein synthesis, inhibition of energy metabolism within microbial cells and inhibition of bacterial efflux pumps.^16,105^ Similarly, antifungal properties may be exhibited by trapping fungal spores in mucilage or wax secretions or preventing germination through secretion of sesquiterpenes amongst other physiological processes.^16^

Among the reported plant's secondary metabolites; antimicrobial mechanisms of alkaloids is thought to be through inhibition of bacterial cell wall synthesis, change in cell membrane permeability, inhibition of bacterial metabolism, and inhibition of nucleic acid and protein synthesis.^83^ Different mechanisms for antimicrobial activity of polyphenols are also reported. These includes enzyme inhibition by the oxidized compounds, possibly through reactions with proteins through SH^−^ groups or through nonspecific interactions. Furthermore, some phenolics such as quinones act as a source of stable free radicals and bind irreversibly with microbial proteins leading to its loss of function. Other targets are inactivating enzymes, binding to adhesins on the microbial cell surface, binding to cell wall proteins, and interacting with substrates rendering them unavailable to the microorganism, complexing with metal ions and others.^86^ In another work, plant flavonoids rich materials are also reported to inhibit bacterial growth by increasing bacterial cell membrane permeability, reduction of ATP production, lowering mobility, and stimulation of host immune system.^80^

### 3.5 Enzyme-based ATFs

Antimicrobial enzymes are other important types of bioactive molecules that can be used to combat microbial infections. They can be incorporated or grafted into polymers or on the polymers surface to prevent microbial colonization.^106^ Enzymes are also used in the development of nanobiocomposite ATFs with nanomaterials.^107^ Recent researches show the fabrication of biomimetic antimicrobial nano-enzymes, called nanozymes.^108^ Nanozymes are immerging as potential substituents to scares natural enzymes, especially for industrial scale applications.

Some antimicrobial enzymes like antimicrobial peptides are reported to be found in natural defensive (immune) system of wide range of organisms including plants.^109,110^ Leitgeb *et al.*,^111^ for example, confirmed the presence of enzymes such as α-amylase, cellulase, lipase, peroxidase, protease, and transglutaminase in the *Aloe arborescens* and *Aloe barbadensis* ethanolic extract. The obtained enzymatic fractions are reported to have microbial growth inhibition activity.^111^

**Fig. 10:**
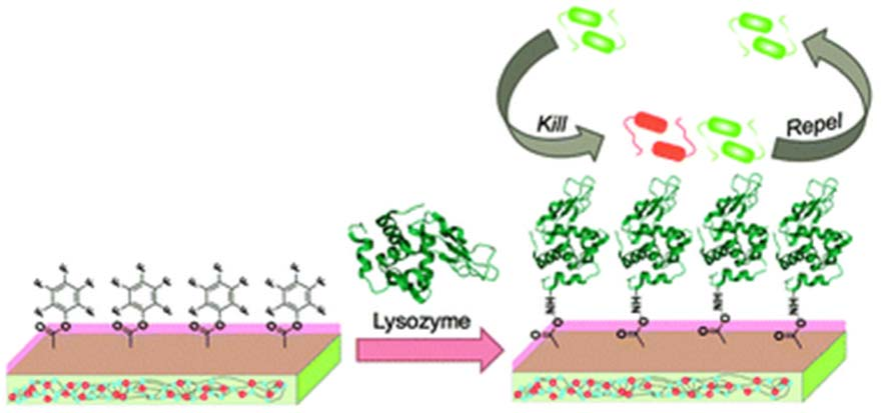
Nucleophilic substitution of lysozyme and bactericidal action of lysozyme based thin film (reproduced from ref. 112).

Lysozyme enzyme deposited pyridinium-based zwitterionic copolymer antibacterial thin film with strong antibacterial and antifouling activity has been reported. The film is prepared by depositing lysozyme onto poly(4-vinylpyridine-*co*-pentaflurophenyl methacrylate-*co*-divinyl benzene) surface by nucleophilic substitution of the pentafluorophenyl group and lysozyme enzyme which result amide bond formation between them.^112^ The surface nucleophilic substitution of lysozyme and bactericidal action of lysozyme based thin film is as shown in Fig. 10.

In another work, Solanki K. *et al.*^113^ developed listeria bacteriophage endolysin activated silica nanoparticles thin film observed to inhibit microbial growth. The covalently bonded thin film is prepared by copolymerization between endolysin and surface modified silica nanoparticles in the presence of polyethylene glycol dimethacrylate as a fixing agent.

Antimicrobial enzymes acts by different mechanisms; some of which include disrupting the cell membrane, degrading proteins and DNA, inhibiting growth, and inducing antibacterial responses from the host.^106^ Among mechanistic action of different class of antimicrobial enzymes, Blackman *et al.*^114^ reported that proteolytic enzymes act as antibiofilm by degrading essential microbial proteins and peptides, polysaccharide depolymerases by destructing the microbial polysaccharides and DNA, quenching enzymes by interfering cell–cell signalling, proteases and nucleases by degrading the extra polymeric substances secreted by the biofilm. Baek *et al.*^115^ confirmed that the mechanism of action of antimicrobial peptides (specifically cecropin P1, antibacterial peptide isolated from the pig stomach) is through electrostatic interaction of positively charged peptides ends (C-terminal) and lipopolysaccharide, which is the main component of the outer membrane of Gram-negative bacteria.

**Fig. 11:**
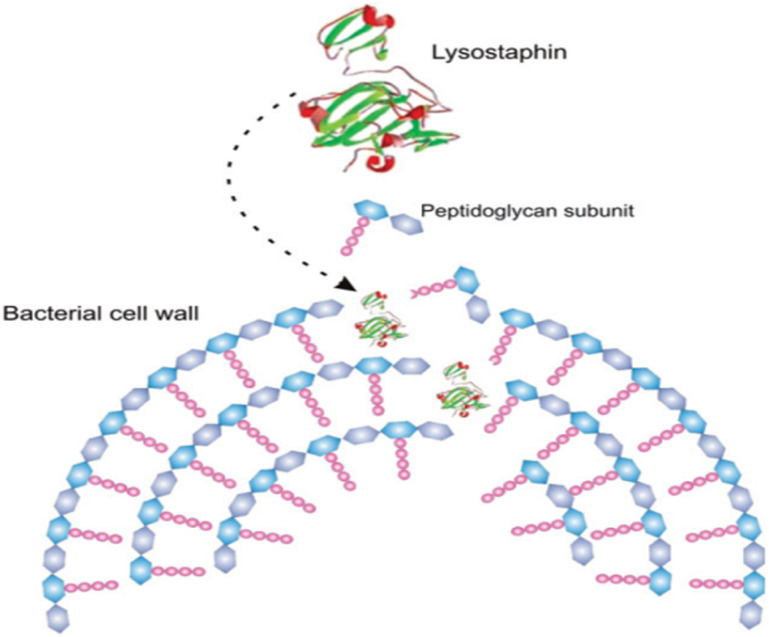
Simplified scheme of the hydrolysis of bacterial cell walls by lysostaphin (reproduced from ref. 106 with permission from John Wiley and Sons).

Furthermore, the same enzymes having different structure acts differently against suspected microbes.^106^ For example, two of protease enzyme subtilisins and lysostaphin inhibit microbial growth through different mechanism; subtilisins hydrolyse bacterial proteins essential for attachment onto solid supports and other bacteria whereas lysostaphin cleaves bacterial cell walls on the third and fourth glycine residues of the pentaglycine cross-bridge. Another class of antimicrobial enzyme, polysaccharide-degrading enzymes, attack bacteria by hydrolyzing 1,4-beta-linkages in the bacterial cell walls. Oxidative enzyme acts by generating ROS against invading pathogens. Fig. 11 shows antibacterial mechanisms of protease enzyme lysostaphin.

### 3.6 ATFs from organic acids

Organic aids are among widely used antimicrobial agent especially in food industry and they can be originated from oxidation of alcohols, fruits, plants and microorganisms among others.^116,117^ Lactic acid, malic acid, propionic acid, tartaric acid, succinic acid, acetic acid, citric acid, fumaric acid, butyric acid and caprylic acid are among the commonly used organic acids.^116^

Previously, natural organic acids decorated zein edible active packaging has been reported with improved antimicrobial activity and flexibility. The film is developed following uniform dispersion of lactic acid, malic acid and tartaric acids in zein solution.^118^ Rocha M. *et al.* also reported sorbic acid and bezoic acid incorporated argentine anchovy protein isolate (API) thin film. API as a film matrix, glycerol as a plasticizer and antimicrobial sorbic acid (SA) or benzoic acid (BA) is mixed prior to homogenization. The film forming solution is poured onto Petri dish and dried to result API-AS and API-BA antimicrobial thin film. The prepared API-organic acids thin film demonstrated strong antibacterial activity against the tested bacteria, escherichia coli, salmonella enteritidis and listeria monocytogenes.^119^

In another work, Suganthi *et al.*^120^ reported PVA polymer film functionalized with different organic acids for food packaging application. Their result confirmed that PVA-malic acid; PVA-tartaric acid and PVA-lactic acid containing packaging films showed better zone of inhibition against *S. aureus* and *E. coli* bacteria when compared with bare PVA film. Romainor *et al.*^121^ also synthesised effective antimicrobial food packaging film from premixed starch, PVA, and citric acid using solution casting method. In contrast to unmodified starch polymer film, the prepared starch-citrate packaging film demonstrated complete growth inhibition against test microorganisms (pathogenic food borne bacteria; *Salmonella thypimurium*, *E. coli*, and *Listeria monocytogenes* and food fungus; *Aspergillus* species, and *Rhizopus* species) and thus enhanced shelf life of some foods under study.

Mechanistic antimicrobial action of organic acids are ability to penetrate into bacterial cells^122^ due to their lipophilic properties which allow them to easily penetrate into microbial plasma membrane, which in turn cause lowering the pH of the medium. Such change in cell medium cause alteration in proteins and phospholipid structures of microbial cell membrane followed by cell growth limit and death.^116,117,123^

**Fig. 12:**
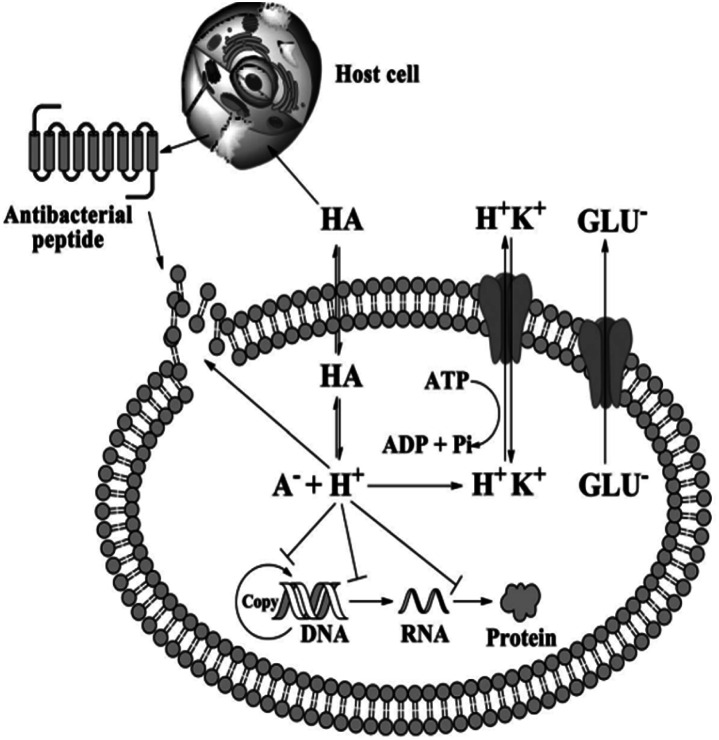
Antimicrobial mechanisms of organic acids (reproduced from ref. 124 with permission from John Wiley and Sons).

The detailed mechanism behind the lowering of the cell pH is that; as organic acids penetrate bacterial cell membrane they undergo dissociation at pH neutral, thus H^+^ produced and intracellular pH decreased.^121,124^ The decreased intracellular pH leads to the protonation of the carboxyl and phosphate groups of lipopolysaccharides on the bacterial cell membrane, thus cell stability disturbed and cause inhibited cell growth. Second, the lower intracellular pH also affects the enzymatic activities and inhibits DNA replication and transcription as well as protein expression. To stabilize this intracellular pH, bacteria must release hydrogen ions *via* active transport, but this process consumes adenosine triphosphate (ATP) and affects the normal growth of bacteria.^124^Fig. 12 demonstrate the general antimicrobial mechanisms of organic acids.

Suganthi *et al.*^120^ reported another possible antibacterial mechanisms of organic acid-based film are; generation of reactive oxygen species, deposition of carboxyl groups on the surface of the bacteria, and tuning of the film surface to prevent bacterial surface adhesion. Organic acids are also reported to enhance bactericidal activity of the materials through increasing the osmotic stress of the cell and disrupting the biomolecule synthesis by releasing their anion and proton, reduction of ATP production by uncoupling electron transport, and disturbance of regulation of bacterial signalling pathways; finally, these all mechanistic cause microbial death.^125^

### 3.7 Bacteriocin-based ATFs

Bacteriocin is bacterial peptide produced by bacterial ribosome which is used as antimicrobial agent.^126^ Like other antibacterial agents they can be incorporated into edible polymers such as thin film to enhance controlled and continuous flow of active agents to maintain high concentration for long period^127^ with additional the advantages that Bacteriocin are stable at higher temperature, work over wide pH range, and have lower minimum inhibitory concentration (MIC) when compared with plant extracts and the other protein-based antimicrobial preservatives.^128^

For instance, antimicrobial thin film from *lactobacillus sakei* extracted bacteriocin and polysaccharide (agarose and agaropectin) as a biopolymer matrix has been prepared and demonstrated strong antibacterial activity. The film is prepared by homogenization of film forming solution followed by casting technique to obtain uniformly dispersed bacteriocin in polysaccharide mixture matrix.^129^ In another work, divergicin M35 (bacteriocin from carnobacterium divergens M35strain) based thin film has been prepared by incorporating the bactriocin in chitosan support matrix. This prepared thin film showed high antibacterial activity due to its synergetic effect with chitosan.^130^

La Storia A. *et al.*^131^ reported three dimensional thin films from whey protein matrix and *Lactobacillus curvatus* 54M16 bacteriocin using gelatin and inuline to form gels and to create synergy among the components to enable the formation of three dimensional networks. High zone of inhibition is observed for bacteriocin incorporated thin film when compared with the control. P. Bagde and V. Nadanathangam^132^ also developed antimicrobial film from Bacteriocin (antibacterial peptides which is extracted from the lactic acid bacteria) immobilized crystalline nanocellulose (BIN) and corn starch. As reported in this work, all the films containing BIN revealed inhibited growth against pathogenic *S. aureus, E. coli* and this growth inhibition comes from the incorporated antibacterial peptides.

**Fig. 13:**
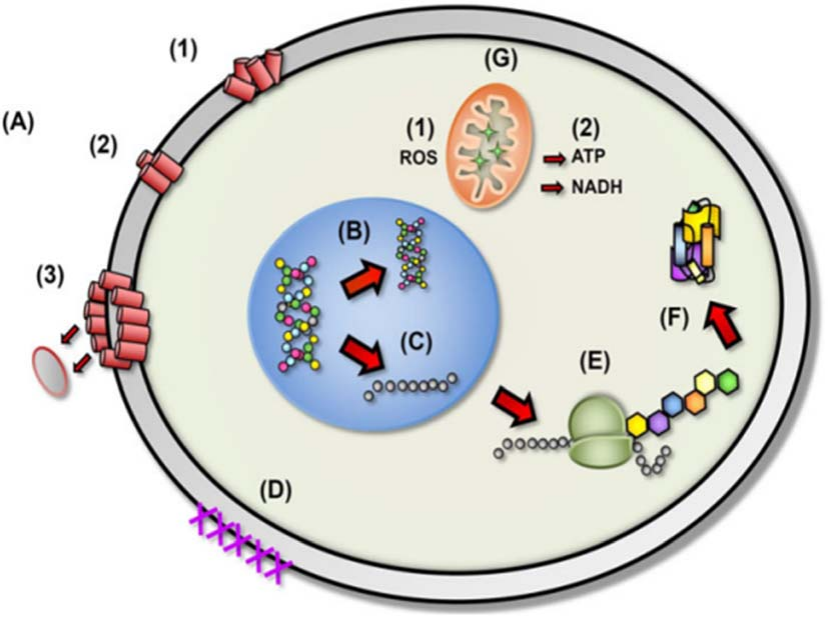
Diverse mechanistic modes of action for antimicrobial bacteriocin in microbial cells (reproduced from ref. 132, used under Creative Commons CC-BY license).

The general mechanism of bacteriocin (antimicrobial peptides) is through disruption of anionic bacterial cell membrane due to the electrostatic forces between positively charged amino acids and the negatively charged cell surface. Fig. 13 demonstrates the detailed bactericidal mechanism of bacteriocin; (A) disruption of cell membrane integrity: (1) random insertion into the membrane, (2) alignment of hydrophobic sequences, and (3) removal of membrane sections and formation of pores. (B) Inhibition of DNA synthesis. (C) Blocking of RNA synthesis. (D) Inhibition of enzymes necessary for linking of cell wall structural proteins. (E) Inhibition of ribosomal function and protein synthesis. (F) Blocking chaperone proteins necessary for proper folding of proteins. (G) Targeting mitochondria: (1) inhibition of cellular respiration and induction of ROS formation and (2) disruption of mitochondrial cell membrane integrity and efflux of ATP and NADH.^133^

### 3.8 Polymer-based ATFs

The development of advanced nanomaterial offers multiple opportunities in a variety of engineering applications including biomedical ones.^134^ Polymer-based anti-microbial thin film is one such example which is gaining immense importance lately since they can act as a physical barrier to prevent the growth, spread or entry of microorganisms like bacteria and fungi into the critical parts inside a device or substrate.^135^ The increasing demand for products that prevent microbial contamination has made polymer-based antimicrobial thin film an attractive option due to its advantages like flexibility, strength and durability as well as its ability to sustain antifungal protection for prolonged periods without compromising performances.^136^

Polymer-based antimicrobial coating design usually consists of three components namely; polymer backbone, adhesive layer and active layer.^137^ Through a variety of surface treatment techniques, the physical and chemical states of the substrate have been induced in order to investigate the mechanism of how the substrate state affects the adhesive. The adhesive layer helps in adhering the film while active layers such as lysozyme, chitosan *etc.*, impart antibacterial/antifungal property by specific killing mechanisms like enzyme blockade.^138^

**Fig. 14:**
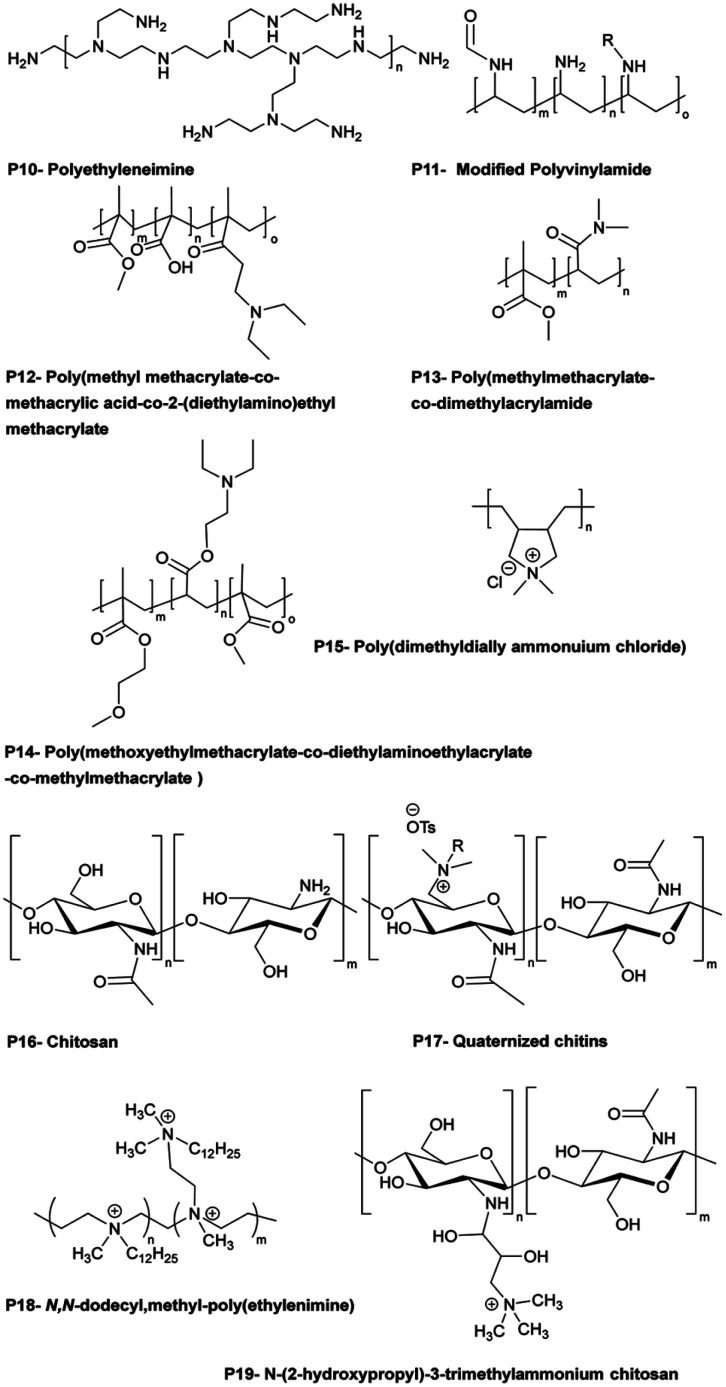
Examples of bactericidal polymers used in layer-by-layer films and as antibacterial coatings (reproduced from ref. 139 with permission from American Chemical Society).

Polymer materials may demonstrate intrinsic antimicrobial activity in the ATF systems or they can serve as a matrix media to accommodate other active agents. Some polymers with intrinsic antimicrobial activity are shown in Fig. 14.^139^ In the latter case, polymers serve as slow release nanocomposite vehicles for the delivery of metal ion or other active agents.^140,141^

For example, antimicrobial polydopamine layer decorated with Zn@CuO under ultrasonication treatment has been developed through complexation and coordination formation between polydopamine and metal oxides. The synergy between the polymer matrix and metal oxides brought about increased bactericidal effect of the developed thin film.^140^ Another illustration is the development of a regenerable silver slow-releasing thin film nanocomposite membrane.^142^ As carriers for Ag^+^ or Ag^0^, nanozeolites were covalently bound to the surface of a polyamide nanofiltration membrane. The findings have shown that the slow release of biocides from porous nanoparticles can act as a long-lasting mechanism for bio-fouling control.

**Fig. 15:**
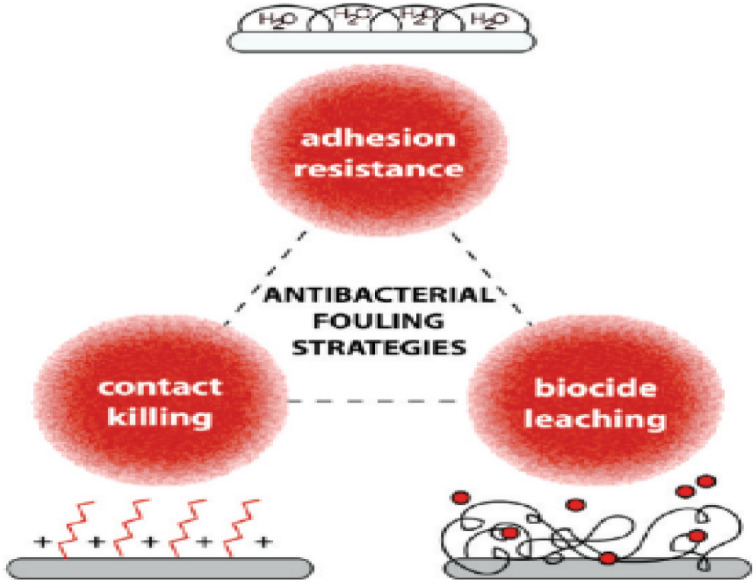
Polymer-based thin film antimicrobial mechanisms (reproduced from ref. 143 with permission from American Chemical Society).

Polymer-based antimicrobial thin films act through three main mechanisms^143^ shown in Fig. 15; physical barriers (non-leaching), contact killing (leaching), and diffusion killing (semi-leaching). Physical barriers prevent bacterial attachment by using contact angles and surface roughness characteristics, while contact killing biofilms provide direct killing by leachate release often *via* ion exchange reactions when dampened and illuminated. Lastly, diffusion killing systems rely on preformed pores and capillaries within the film matrix that enable small amounts of chemicals scattered throughout the layer to slowly oozes out using thermally induced polymer chain motions over time upon exposure to humidity or light triggers contributing localized & prolonged efficacy while reducing transport away from site (reducing environmental impact).^144^

In addition to the above classes of nano-assisted materials, there also exist other emerging antimicrobial materials such as quantum dots,^145^ graphene oxides,^146^ metal sulphides,^147^ and MXenes,^148^ which are reviewed elsewhere. As discussed in this review, these nanostructured materials are also used as modifiers or functionalizing agents of other nanomaterials and nano-assisted thin films for synergetic antimicrobial properties.

## 4. Summary and future outlook

Interactions between surface treatment methodologies continues to play a fundamental role in today's product development process – hence having solutions such as antimicrobial thin films will prove beneficial in this regard in providing effective prevention against microbial contamination. While traditional approaches continue to remain relevant today, there is no doubt that antimicrobial thin film technology has come a long way over recent years owing its success primarily due its high efficacy levels combined with fewer risks associated with its use when compared with chemical solutions typically used for similar purposes. Thin films possess both physical and chemical properties which contribute to their biocidal action; antimicrobial thin films often contain small particle sizes that can physically disrupt bacterial cell walls or act as physical barriers, preventing microbial attachment and proliferation on treated surfaces or chemical biocides primarily penetrate cell membranes by oxidation reaction or simply by dissolving into cells *via* electrochemical transduction processes and cause microbial cell damage.

The robust nature of thin films allows the development and scale up of practical nano-assisted antimicrobial systems by immobilizing various active ingredients, including metals, metal oxides, plant bioactives, enzymes, organic acids, bacteriocins and polymers. Accordingly, these systems pave the way for the realization of large scale advanced antimicrobial systems for applications like wound dressings, tissue engineering, membrane-based water treatments, active food packaging, surface disinfection, self-healing surfaces, blocking of UV and other damaging radiations. The multitude options in design, development and large-scale processing of nanostructured antimicrobial thin films still provides multitude of research innovation opportunities to realize affordable and efficient treatment systems.

## Author contributions

Bilisuma Finina wrote the main manuscript. Anteneh Mersha conceptualized the idea, reviewed and edited the manuscript.

## Conflicts of interest

There are no conflicts of interest to declare.

## Supplementary Material
